# From dynamic chromatin architecture to DNA damage repair and back

**DOI:** 10.1080/19491034.2017.1419847

**Published:** 2018-01-30

**Authors:** Emmanuelle Fabre, Christophe Zimmer

**Affiliations:** a Equipe Biologie et Dynamique des Chromosomes, Institut Universitaire d'Hématologie, Hôpital St. Louis, Paris, France; b CNRS, UMR 7212 INSERM U944, IUH, Université Paris Diderot Sorbonne Paris Cité, Paris, France; c Institut Pasteur, Unité Imagerie et Modélisation, 25 rue du Docteur Roux, 75015, Paris, France; d UMR 3691, CNRS; C3BI, USR 3756, IP CNRS, Paris, France

**Keywords:** homologous recombination, chromatin dynamics, chromatin structure, Polymer simulations, nuclear organization, Chromatin, Chromosome, DNA repair

## Abstract

Maintaining the integrity of the genome in the face of DNA damage is crucial to ensure the survival of the cell and normal development. DNA lesions and repair occur in the context of the chromatin fiber, whose 3D organization and movements in the restricted volume of the nucleus are under intense scrutiny. Here, we highlight work from our and other labs that addresses how the dynamic organization of the chromatin fiber affects the repair of damaged DNA and how, conversely, DNA damage and repair affect the structure and dynamics of chromatin in the budding yeast nucleus.

## DNA damage repair by homologous recombination

**Figure 1.**
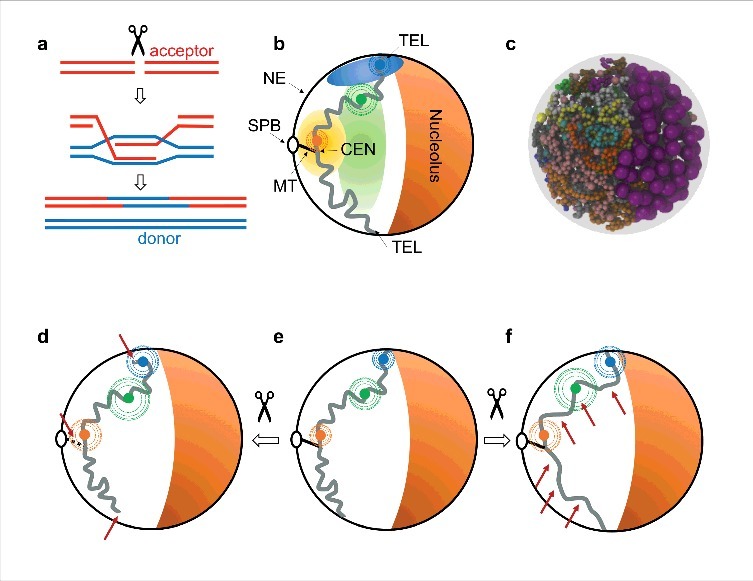
Links between chromatin organization and DNA damage and repair. (a) Simplified schematic of homologous recombination (HR), where a broken DNA double strand (acceptor, red, top) uses an intact homologous sequence (donor, blue) as a template for repair. The process involves degradation of the 5′ ends at either side of the DSB by nucleases, forming single stranded DNA intermediates (resection, not shown), invasion of the donor by the acceptor's single DNA strands via base pairing of the complementary sequences, followed by DNA synthesis (center), DNA ligation and results in two intact DNA double strands (bottom). The process that brings the donor and acceptor sequences in contact is known as homology search. (b) Simplified schematic of the Rabl-like chromosome organization in budding yeast. A single chromosome is shown (grey curve). The centromere (CEN) is linked through the kinetochore complex (not shown) and a single microtubule (MT) to the spindle pole body (SPB). The telomeres (TEL) are tethered to the nuclear envelope (NE). The nucleolus occupies a membrane-less, crescent-shaped compartment opposite to the SPB. Individual loci (orange, green and blue disks) are statistically confined to subnuclear ‘gene territories’ (pale orange, green and blue regions) and undergo subdiffusive movements (dashed circles), with reduced mobility for loci near centromeres or telomeres. (c) Computational simulations of chromosomes based on polymer physics can successfully predict many observed features of yeast chromosome organization, as determined by imaging and Hi-C data. A snapshot of a Brownian dynamics simulation is shown (reproduced from [41] with permission from BioMed Central). (d,e,f) Two possible causes of enhanced chromatin dynamics following DNA damage by DSBs: relaxation of the link between centromere and SPB and untethering of the telomeres from the NE [54,58] (d, red arrows), or stiffening [22,55] (or alternatively, decondensation [21]) of the chromatin fiber (f, red arrows).

Among the most lethal types of damage to the genome are breaks to both strands of the DNA double helix (double strand breaks, DSB). Genomes have evolved elaborate molecular mechanisms to repair these lesions. The two main pathways for DSB repair are non homologous end joining (NHEJ) and homologous recombination (HR). In NHEJ, the two extremities resulting from the DSB are simply rejoined together, after a limited enzymatic processing that can lead to error-prone repair, and hence mutations. In HR, the dominant repair pathway in yeast, the genetic information on an intact homologous DNA template is used to resynthesize the original DNA sequence at the broken site, thereby allowing error-free repair [1] (Fig. 1a). This process requires a physical contact between the intact template (donor sequence) and the broken site (acceptor sequence). The availability of one or more homologous donor sequences depends on whether the genome is haploid or diploid, and on the cell cycle phase: in diploid cells, the homologous chromosome provides a donor during the entire cell cycle; in haploid cells (which we subsequently focus on), a copy of the broken chromosome is available only during S/G2 phase, in the sister chromatid. In budding yeast, sister chromatids remain closely associated to each other by cohesion until the transition from metaphase to anaphase. In G1 phase, where the sister chromatid is not available, HR may occur with a homologous sequence located elsewhere in the genome, potentially leading to chromosome rearrangements. In budding yeast, this is rarely the case aside from repeated regions such as telomeres or the ∼1 Megabase long array of rDNA genes or the mating type loci. However, for experimental studies of HR, one can genetically insert artificial homologous donor and acceptor sequences at distinct and specified locations in the yeast genome, and use inducible endonucleases to target a cleavable sequence inserted in the acceptor site, thereby inflicting DSBs at this location [2–4]. In these ectopic recombination experiments, one can therefore control not only where the DNA damage occurs, but also with which donor sequence it will undergo successful HR. In addition, it is possible to measure the efficiency of the repair process, for example by comparing the growth of cells with and without induction of endonucleases or by monitoring molecular recombination products with PCR. Experimental systems of this type have allowed geneticists to study the mechanisms of HR in great detail, and to dissect the molecular pathways that take place before and after the homologous sequences are brought into contact [1]. However, central but less studied questions are how these small homologous sequences come into contact in the first place in the comparatively vast space of the nucleus, and whether and how the process of homology search that brings the sequences in contact affects the outcome of HR [5]. An apparently straightforward case is that of sister chromatids in G2, where the intact donor is in immediate proximity to the broken acceptor sequence. However, in G1, where the homologous sequence, if available, is located elsewhere in the nucleus, HR requires a movement of at least one of the sequences relative to the other. It is therefore important to understand the mechanism of homology search and how the spatial chromosome organization might affect the repair process. Before highlighting studies that shed light on these questions, we briefly summarize what is known about the 3D organization and dynamics of chromatin in the budding yeast nucleus.

### Spatial organization of yeast chromosomes

In haploid budding yeast, roughly 12 Megabases of DNA are distributed in 16 chromosomes and packed in a nucleus of approximately 2 micrometers in diameter [6,7]. Much is known about the spatial organization and dynamics of yeast chromatin, thanks to a number of imaging and chromosome conformation capture experiments and several modeling studies (Fig. 1b). Imaging studies have shown that each centromere is tethered to the spindle pole body (SPB) via a single nuclear microtubule and the kinetochore complex, and that telomeres are tethered to the nuclear envelope (NE). In combination with automated image analysis, live cell microscopy has been used by us and others to map nuclear territories occupied by individual chromatin loci [8,9]. Although the subnuclear position of any given locus can vary strongly from cell to cell, statistical mapping reveals preferential positioning in certain regions of the nucleus, which we called ‘gene territories’, in loose analogy with the chromosome territories observed in higher eukaryotes [10]. The approximate location of each territory is largely determined by chromosome arm length and exclusion from the nucleolar compartment (which contains the rDNA array) [8,11,12]. The conformation of budding yeast chromosomes emerging from these and related studies is reminiscent of that first described by Carl Rabl in nuclei of salamander cells, which display a polar arrangement of centromeres and telomeres on opposite sides of the nucleus [13]. We note that in addition to this generic organization of chromosomes, many reports have analyzed changes in positioning of individual genes (typically, relocation to the NE, often involving interactions with nuclear pores), in a manner that depends on the presence of specific DNA sequences (‘zip-codes’) [6,7,14].

### Chromatin dynamics and contact frequencies in yeast

Despite the frequent use of the word ‘architecture’, the organization of chromatin in the nucleus is far from static. Time-lapse microscopy observations have been used early on to track the movements of individual fluorescently tagged loci in live cells [15–17] and more recent studies have prolonged these efforts using improved imaging technology [18–22]. These movements appear to be mostly or exclusively stochastic. Although some reports have described directed movements of chromatin loci in mammalian cells [23,24], to our knowledge such movements have not been unequivocally demonstrated in interphase yeast. Stochastic movements are generally analyzed by computing mean square displacements (MSDs) as function of time interval. For free diffusion (e.g. for particles undergoing Brownian motions), MSDs increase linearly with time, and the slope provides the diffusion coefficient. By tracking loci over periods of up to several minutes with images taken every few seconds, initial studies have mostly described chromatin dynamics as free diffusion in confined volumes. Estimated diffusion coefficients ranged from ∼5×10^−4^ μm^2^/s to ∼7×10^−3^ μm^2^/s, and radii of confinement to subnuclear regions from ∼0.4 μm to ∼0.75 μm, depending on ploidy, growth conditions (such as the carbon source), locus position on the chromosome arm (with centromeric and telomeric regions exhibiting more constrained movements), and cell cycle phase (with more confined movements in S phase compared to G1) [15,16,25]. More recent analyses and experiments, some of which used imaging rates of up to 100 frames per second, have revealed that chromatin actually undergoes subdiffusion, i.e. the MSD increase significantly slower than expected for free diffusion, suggesting that locus movements are restricted by more than a confining region. The increase of MSD with time has been characterized by power laws with exponents ranging mostly from ∼0.4 to ∼0.7 [17–20,22,9].

What drives and restricts these stochastic movements ? A definitive answer to this question is lacking. However it is now clear that the motion of a single locus cannot be understood in isolation from the rest of the chromosome, and the simplest polymer dynamics model (the Rouse model), which treats monomers as beads connected by springs, predicts a subdiffusion exponent of 0.5 on some time scales, in at least rough agreement with observations [18,26]. Nevertheless, other factors might also be at play, such as viscoelasticity of the nucleoplasm [27]. The apparent absence of directed motions may seem to support a purely passive, thermally driven motion and argue against the direct involvement of molecular motors in chromatin dynamics. However, early studies observed reduced dynamics upon ATP depletion [15,28] and experiments in bacteria and yeast support a role of ATP consuming enzymes, possibly chromatin remodeling enzymes, in driving stochastic chromatin movements [29]. Recently, two studies highlighted the influence of the cytoskeleton in chromatin dynamics. Spichal et al. showed a role of both cytoplasmic and nuclear actin on locus dynamics [30], acting via chromatin remodeling enzymes and possibly transcription, while Lawrimore et al. observed defects in chromatin mobility upon experimental disruption of microtubules by Nocodazole [31]. The precise molecular mechanisms that power chromatin dynamics in addition to mere thermal agitation remain to be fully understood.

Chromatin dynamics has direct implications on the homology search. Despite the restricting effect of the chromosome on the motion of single loci, observations indicate that most loci can explore a sizeable fraction of the nucleus within minutes [16,17]. Therefore, it seems possible (but remains to be demonstrated) that any locus in the genome might contact any other locus within a single cell cycle. Experiments that directly address this question are presently lacking. Genome-wide chromosome conformation capture experiments (Hi-C) have been used to determine DNA-DNA contact frequencies throughout the yeast genome [32–34], but are currently restricted to averages over millions of cells, and do not yet provide absolute contact numbers in single cells. Nevertheless, these studies provide useful quantitative information about contact frequencies between pairs of loci relative to other pairs of loci and revealed strong differences across the genome. For example, genomically proximal loci or centromeric sequences are much more frequently in contact than loci separated by large genomic distances or non-centromeric loci on distinct chromosomes. Before turning to the implications of these contact frequencies on HR, we briefly discuss computational models of chromosomes, whose development has been spurred by Hi-C and which in turn provide useful insights into chromatin structure, as briefly reviewed below.

### Computational models of yeast chromosomes

As we reviewed elsewhere [35,36], efforts to model chromosomes come in two main flavors. First, inverse models use Hi-C and/or imaging data as input to reverse engineer one or more 3D structures compatible with the data. The resulting structures might facilitate the identification of features that are not immediately apparent in the raw data, but do not provide a predictive understanding. By contrast, direct models are built from first principles plus a small set of assumptions and parameters and aim at predicting data such as DNA-DNA contact maps. This second approach has turned out to be remarkably effective for budding yeast [26,37–41]. More specifically, we and others showed that a polymer simulation that models Brownian dynamics of polymer chains with the inclusion of just a few sequence-specific constraints (tethering of centromeres to the SPB and of telomeres to the NE, plus specific assumptions about the nucleolar rDNA) can recapitulate a large amount of experimental measurements including genome-wide contact frequencies from Hi-C and gene territory positions or average distances between loci determined by imaging [26,37,38] (Fig. 1c). Our model requires assumptions of some key parameters of the semi-flexible chromatin fiber, in particular the compaction (the number of base pairs per unit length) and the persistence length (the distance over which the polymer chain can bend due to thermal motions alone), which can be treated as two independent parameters. In a recent study, we systematically varied compaction and persistence length parameters in simulations of all chromosomes in the yeast nucleus, and compared the model predictions to a wide range of experimental data, including Hi-C and imaging data, gathered from multiple laboratories [41]. Using a Bayesian inference approach we calculated the probability density of the model parameters based on the experimental data. This method allowed us to place new bounds on the average compaction and persistence lengths of yeast chromatin, namely ∼53–65 bp/nm and 52–85 nm, respectively [41] (providing further evidence against a 30 nm chromatin fiber in this organism [42]). The model is in excellent agreement with Hi-C data and static imaging data and can approximately reproduce the MSD curves of multiple loci as function of time interval (after fitting the simulation time units to the experimental time), suggesting that our model is also able to correctly account for the dynamics of chromatin in vivo [41]. Such polymer models can impact our understanding of DNA repair and HR as we discuss next.

### Effect of chromosome organization on DNA damage repair

How does the dynamic organization of chromatin in the nucleus affect DNA repair by HR ? In order to address this question, Agmon et al. [4] created an ectoptic recombination assay, where pairs of artificial donor and acceptor sequences were introduced at specific positions in the genome. Twenty two pairs of loci were considered, at genomic locations close to centromeres, or to telomeres, where either low or high contact frequencies were expected based on our view of nuclear architecture detailed above [11,32,37] (Fig. 1b). The measured repair efficiencies were found to correlate positively with the contact frequencies determined from imaging experiments [11] or predicted by our simulation [26,37]. This study provided a first demonstration that the homology search through the nucleus is rate limiting for HR, and that differences in repair efficiency among pairs of loci can to some extent be predicted in silico. These findings were confirmed and extended by a subsequent study that considered donor loci at many other positions along multiple chromosome arms, including internal positions far from centromeres or telomeres [43]. Spatial proximity was also found to influence repair efficiency in another recent report [44]. However, the rate of successful DNA repair, as judged by cell survival rates, also depends on other factors identified in these studies. These factors include the extent of sequence homology [43], the rate of 5′ to 3′ DSB end resection [4,43], the abundance of the replication factor A [43], or the presence of repressive chromatin [44]. While deciphering the precise contribution of each of these factors (and potentially others) in the repair efficiency will require follow-up work, these reports all support the notion that the spatial organization of chromatin fibers in nuclear space constrains the outcome of DNA damage and repair.

### Effect of DNA damage and repair on chromatin dynamics

What about the inverse relation, i.e. the effect of DNA damage and repair on chromatin organization ? A DSB might be expected to not only alter the local structure of chromatin, but also to modify chromosome organization globally. In particular, one could imagine that a DSB physically separates the chromosome in two separate and shorter polymer chains with fewer tethering constraints (one chain being freed from telomeric tethering and the other freed from centromeric tethering), which should lead to a drastic change of their spatial configuration and dynamics. However, it was shown that the two new extremities resulting from a DSB are in fact held together, such that the chromosome remains a single connected chain [45–47]. More profound changes to the chromatin result not from the break itself, but from the ensuing cellular response. The DNA damage response (DDR) activates molecules that alter the chromatin in order to recruit the molecular factors necessary for HR. These chromatin changes include posttranslational histone modifications and nucleosome remodeling, and can spread along the chromosome to long distances away from the damaged site, and potentially also to other chromosomes [48–50]. Since the configuration and movements of chromosomes depend in part on the mechanical properties of the chromatin fiber such as its compaction and rigidity [41,51] (see above), these chromatin changes can be expected to alter chromosome organization or dynamics. Indeed, several studies using time-lapse microscopy of chromatin loci have shown that yeast chromatin dynamics is modified after infliction of DNA damage by either induced endonucleases, treatment with the genotoxic drug Zeocin or irradiation with gamma rays [21,52–56]. Note that these methods lead to very different genomic distributions of DSBs: whereas endonucleases inflict local damage by targeting a single locus, Zeocin or gamma rays provoke global damage via an undetermined number of breaks at random locations in the genome. The nature of this change in chromatin dynamics appears to depend on the time-scales investigated. In most studies, chromatin loci were imaged hours after exposure to Zeocin or during continuous induction of endonucleases. Loci were then tracked over periods of up to a few minutes, with temporal resolutions in the range of one second. These studies consistently reported a significant increase in chromatin dynamics upon DNA damage, which was mostly characterized by an increase in the radius of confinement [52–54,57,58]. In our recent study, we tracked loci at 100 ms time intervals over minutes in several hundreds of cells subjected to Zeocin treatment [22]. Analysis of the MSD between 0.1 and 10s indicated an increase in dynamics at all these time scales, and an increase of the subdiffusion exponent to ∼0.65–0.75, corroborating similar findings in a recent study by Hauer et al. [21] We note, however, that a recent study by Mine-Hattab et al. [19] using faster imaging (down to 10 ms), while confirming increased mobility at large time scales, reported a reduction of MSD at small time scales. An earlier study by Saad et al. [56] also observed reduced mobility of a locus adjacent to the broken site, but unlike most other reports focused on the early steps following the DSB, during resection. Nevertheless, for the later stages of the cellular response to DSBs, enhanced chromatin mobility both near the DSB and elsewhere in the genome appears to be consistently observed at time scales ranging from seconds to minutes [22,52–54].

### Possible mechanisms of DNA damage dependent increase in chromatin dynamics

What is the mechanism of this increase in chromatin mobility following DNA damage ? In light of the known and potential factors that influence chromatin dynamics (see above), many distinct causes can in principle apply. Among them, recent work focused on two potential causes: (i) relaxation of the tethering constraints that restrict chromosome movements, in particular at the centromeres and telomeres [54,58,25] (Fig. 1d), and (ii) modifications of the mechanical properties of chromatin that affect its dynamics [19,21,22] (Fig. 1f). By searching for phosphoproteins modified by the checkpoint kinase Mec1 upon DNA damage, Strecker et al. homed in on the Cep3 kinetochore protein, and reported a loosening of the kinetochore-microtubule connection upon Cep3 phosphorylation, thereby relaxing the link between centromere and SPB [54]. An experimentally induced loosening of this link (by activating transcription through the centromeres) as well as an untethering of telomeres to the NE (using the mutant Δsir4 defective for telomere tethering) led to an increase in chromatin mobility without DNA damage similar to that provoked by induced DSBs [54]. More recently, a related study by Lawrimore et al. [58] failed to confirm DNA damage-dependent detachment of centromeres, but also observed detachment of telomeres and showed that mutants with untethered telomeres exhibit a comparable increase in chromatin dynamics to wild type cells exposed to DNA damage. This study further reported an expansion of centromeric chromatin and also implicates the dynamics of the microtubule cytoskeleton in the enhanced chromatin movements. Hauer et al. [21] and Herbert et al. [22] explored the possibility raised earlier [57,59] that the mobility increase could arise from altered chromatin properties. In order to assay chromatin structure in vivo, both studies measured distances between intrachromosomal pairs of loci and observed an increase in these distances upon Zeocin treatment concomitant with enhanced chromatin subdiffusion (with subdiffusive exponents varying from ∼0.65 to ∼0.75) [21,22].

What causes this increase in intrachromosomal distances and how does it relate to chromatin dynamics ? In order to address this, we (Herbert et al. [22]) turned to our previously developed computer simulations [41] (see above) and examined the effect of either increasing or decreasing the global compaction or rigidity of the fiber. We found that only increasing the rigidity of the chromatin (by increasing its persistence length) could simultaneously explain the increase of intrachromosomal distances and enhanced chromatin dynamics, in qualitative accordance with observations. This indirect evidence for chromatin stiffening was corroborated by super-resolution imaging of a fluorescently labeled locus, consisting of an inserted bacterial operator sequence, which revealed a small but significant change in shape more consistent with stiffening than with decondensation [22]. A seemingly opposite conclusion was drawn by Hauer et al., who instead proposed a global increase of chromatin flexibility and decondensation. This was based on the observation of reduced levels of core histones throughout the genome and on a distinct computational analysis of locus trajectories that aims to infer external forces acting on the moving polymer [60]. This analysis, based on a different polymer model and using different biophysical parameters, inferred a reduction of these forces upon DNA damage [21]. Although consistent with a chromatin decondensation, this analysis does not appear to directly address potential changes in chromatin rigidity. By contrast, in an earlier study, Verdaasdonk et al. [25] experimentally depleted H3 histone levels and instead observed a reduction of the volume explored by chromatin loci, which the authors attribute to a diminished persistence length. Although this implies a reduction of bending rigidity, Verdaasdonk et al. [25] described this effect as a stiffening of the chromatin fiber. While unintuitive, this interpretation is based on the entropic spring model, which considers the polymer as a spring that resists forces applied to both ends to stretch it. In this model, the spring stiffness is inversely proportional to the persistence length [61], therefore a fiber that is less resistant to bending (and in this sense more flexible) is harder to stretch (and in this sense stiffer). Finally, we note that the study by Miné-Hattab *et al.* also proposed a global increase in bending rigidity, but on the basis of yet another model [19]. It remains to be seen how these seemingly conflicting observations and modeling approaches can be reconciled. We believe that additional experimental data, for example high resolution images of extended chromatin fibers rather than of single loci, will be critical to shed more light on how chromatin architecture is altered in response to DNA damage.

### Open questions

Many other questions remain to be addressed. One key question is to what degree the effect of DNA damage on chromatin structure extends away from the damaged site to the entire chromosome and to other chromosomes. By targeting endonucleases to specific loci, multiple labs have shown that chromatin dynamics is enhanced away from the damaged site as well as on other chromosomes, albeit less so [22,53,54,57]. Our modeling results and those of Miné-Hattab et al. are consistent with a global stiffening of the chromatin throughout the genome [19,22]. At the experimental level, from experiments using Zeocin, it is hard to discriminate between local effects at the site of the DSB and global effects, because this drug creates an unknown number of DSBs at unknown, presumably random, locations in the genome. Future experiments should use targeted breaks and determine if intrachromosomal distances increase both on undamaged chromosomes and on the broken chromosome. If global chromatin stiffening is confirmed, it remains to be understood how the repair signal propagates from a single DSB to the other chromosomes. One intriguing possibility is that centromeres might provide a platform for such signal propagation owing to the frequent contacts between these chromatin regions in the nucleus.

What molecular mechanism underlay the chromatin stiffening proposed by us and others [19,22] ? As already mentioned, the cellular response to DNA damage involves extensive modifications of the chromatin at the broken site, including posttranslational histone modifications, nucleosome remodeling, and the recruitment of HR proteins. All these factors can potentially be responsible for the changes in chromatin properties. In principle, it is also possible that the reported changes are caused by Zeocin binding to the chromatin directly, rather than the subsequent DNA damage response. However, we provided evidence that phosphorylation of histone H2A, which is known to spread along the chromosome at ∼30 Kb on either side of the break [48], is partly responsible for global chromatin stiffening, because in a phosphorylation defective mutant the damage dependent increase in intrachromosomal distances was reduced [22]. In addition, Miné-Hattab et al. propose that the repair factor Rad51, which forms a nucleofilament together with single stranded DNA at the lesion, leads to rigidification of the chromatin fiber, which is consistent with their observation that the change in mobility is abolished in absence of this protein [19]. Hauer et al. report a genome-wide reduction in core histones following DNA damage by Zeocin and found that an experimentally induced depletion of histones also increased chromatin mobility without DNA damage [21]. On the other hand, Verdaasdonk et al. observed a reduction of locus confinement radii upon depletion of H3^25^. Thus, both H2A phosphorylation and H3 and H4 depletion appear to contribute to structural modifications of chromatin that affect its mobility. It remains however to be understood how these chromatin modifications are related to each other and how their interplay, if any, is regulated.

What might be the functional consequences of increased chromatin mobility on DNA repair ? It is tempting to speculate that the enhanced mobility accelerates the search for homologous partners and thereby increases HR efficiency. In agreement with this notion, Dion et al [52]. observed reduced interchromosomal repair kinetics in a Rad9Δ mutant with reduced mobility of Rad52-GFP repair foci. Conversely, Hauer et al [21]. observed an increase in repair efficiency in conditions of histone loss. On the other hand, Strecker et al [54]. reported that HR efficiency was unchanged in a cep3 mutant that does not exhibit DNA damage dependent increase in chromatin mobility. More work will be needed to resolve these apparently divergent findings.

Finally, more detailed computational modeling of the homology search and of the HR process in the context of 3D chromosome organization will be needed to clarify quantitatively how the structure and dynamics of the chromatin fiber in nuclear space influences DNA damage repair and vice-versa.
